# Cumulative incidence for wait-list death in relation to length of queue for coronary-artery bypass grafting: a cohort study

**DOI:** 10.1186/1749-8090-1-21

**Published:** 2006-08-24

**Authors:** Boris G Sobolev, Lisa Kuramoto, Adrian R Levy, Robert Hayden

**Affiliations:** 1 Department of Health Care and Epidemiology, University of British Columbia, Canada; 2 Centre for Clinical Epidemiology and Evaluation, Vancouver, Canada; 3 Centre for Health Evaluation and Outcome Sciences, Vancouver, Canada; 4 British Columbia Cardiac Registries Surgical Research Committee, Vancouver, Canada

## Abstract

**Background:**

In deciding where to undergo coronary-artery bypass grafting, the length of surgical wait lists is often the only information available to cardiologists and their patients. Our objective was to compare the cumulative incidence for death on the wait list according to the length of wait lists at the time of registration for the operation.

**Methods:**

The study cohort included 8966 patients who registered to undergo isolated coronary-artery bypass grafting (82.4% men; 71.9% semi-urgent; 22.4% non-urgent). The patients were categorized according to wait-list clearance time at registration: either "1 month or less" or "more than 1 month". Cumulative incidence for wait-list death was compared between the groups, and the significance of difference was tested by means of regression models.

**Results:**

Urgent patients never registered on a wait list with a clearance time of more than 1 month. Semi-urgent patients registered on shorter wait lists more often than non-urgent patients (79.1% vs. 44.7%). In semi-urgent and non-urgent patients, the observed proportion of wait-list deaths by 52 weeks was lower in category "1 month or less" than in category "more than 1 month" (0.8% [49 deaths] vs. 1.6% [39 deaths], *P *< 0.005). After adjustment, the odds of death before surgery were 64% higher in patients on longer lists, odds ratio [OR] = 1.64 (95% confidence interval [CI] 1.02–2.63). The observed death rate was higher in category "more than 1 month" than in category "1 month or less", 0.79 (95%CI 0.54–1.04) vs. 0.58 (95% CI 0.42–0.74) per 1000 patient-weeks, the adjusted OR = 1.60 (95%CI 1.01–2.53). Longer wait times (log-rank test = 266.4, *P *< 0.001) and higher death rates contributed to a higher cumulative incidence for death on the wait list with a clearance time of more than 1 month.

**Conclusion:**

Long wait lists for coronary-artery bypass grafting are associated with increased probability that a patient dies before surgery. Physicians who advise patients where to undergo cardiac revascularization should consider the risk of pre-surgical death that is associated with the length of a surgical wait list.

## Background

In patients with coronary artery disease (CAD) who are to undergo coronary artery bypass grafting (CABG), delaying that operation may lead to the deterioration of the patient's condition, a poor clinical outcome, and an increased risk of death [1-3]. A patient who presents with the symptoms of CAD is usually referred to a cardiologist, who evaluates the results of coronary angiography and recommends treatment. If coronary angioplasty is not indicated, that patient is referred to a cardiac surgeon, who assesses the need for and suitability of CABG surgery. Patients who require immediate care are admitted to a hospital cardiac ward directly from the catheterization laboratory. Elective patients are scheduled for outpatient consultation with the cardiac surgeon. After the consultation in which a CABG is deemed necessary, surgeons register patients on their wait lists. The detailed pathway to surgical revascularization has been described elsewhere [4]. Surgical wait lists hold patient names until surgery can be scheduled. Patients are removed from the wait list without having undergone surgery if they die, refuse the operation, accept surgery from another surgeon, move out of the province, or experience a health-related decline that contraindicates surgery.

It has been argued that cardiologists and their patients should assess the likely extent of treatment delay and associated risks when they choose a cardiac surgeon [5]. In deciding where to undergo treatment, wait-list size is often the only information available to cardiologists and their patients because the length of the wait list for surgery is a common correlate of the expected wait for hospital admission. Indeed, all patients on a wait list must be treated before a patient who has just registered for surgery can be scheduled for treatment. We previously performed an empirical analysis of a population-based registry and found that the length of queue at registration affected the time to elective surgery [4]. Surprisingly, few studies have correlated the health effects of the pre-surgical wait with wait-list size at the time of registration for an elective CABG. The common concern is whether the decision to refer a patient to a specific cardiac surgeon can be made without considering the length of the current wait list.

We performed a prospective study of all patients who registered to undergo isolated CABG surgery from 1991 through 2000 in British Columbia, Canada. We estimated the time-dependent probability for death during or before a certain wait-list week in a patient who could be removed from a surgical waiting list for surgery, death, or other reasons. The objective of this study was to compare the cumulative incidence of wait-list death between two groups of patients classified according to the length of wait lists at the time of their registration for CABG and to test for significant differences in the risk of death resulting from registration on a longer wait list.

## Patients and methods

### Data sources

The data were taken from the British Columbia Cardiac Registries [6]. That prospectively collected database contains information about registration, procedure, or withdrawal dates, and about disease severity and other risk factors for all patients registered for surgical coronary revascularization in 1 of the 4 tertiary-care hospitals that provide cardiac care to adult residents of the Canadian province of British Columbia since 1991 [4]. To identify the date and underlying cause of death of registered patients who died before they could undergo CABG, we linked the registry to British Columbia Linked Health Database Deaths File by patients' Provincial Health Number [7]. Underlying causes of death were coded according to the International Classification of Diseases, 9th revision (ICD-9). To identify coexisting medical conditions in the study cohort we linked the registry to the BC Linked Health Database Hospital Separations File [8] for the period of 1990 through 2001 and retrieved diagnoses reported in discharge abstracts within 1 year before registration for CABG [9]. The University of British Columbia Ethics Board approved the protocol for this study.

### Patients

Between January 1991 and December 2000, 9366 records of patients who registered for isolated CABG were added to the registry. We excluded 30 records of patients who were coded as emergency cases, 99 who had the same date for registration and removal, 4 whose operating room report was missing, and 267 who underwent surgery within one to three days after having been registered on a wait list. The remaining 8966 records had either the surgery date or the date and reason of removal from the wait list without surgery. Because patients whose angiographic findings indicated the need for immediate surgery were not added to a wait list, they were not included in the analysis of wait-list mortality but instead contributed to demand for service figures.

### Urgency groups

When accepting patients on wait lists for CABG in British Columbia, all cardiac surgeons use a common guideline to indicate the priority for booking the operating room according to the patient's anginal symptoms, coronary anatomy, and left ventricular function so that surgery can be performed within a clinically appropriate time [10]. In this analysis, patients are classified as "urgent" if the suggested time to surgery was 3 days after the treatment decision had been made, "semi-urgent" if that time was 6 weeks, or "non-urgent" if that time was 12 weeks.

### Demand for surgery

For each calendar week during the study period, the demand for surgery was characterized by the size of existing wait lists and by the number of direct admissions, i.e., patients admitted to a hospital ward immediately after angiography. For each patient, the wait-list size at registration was a count of patients with higher or equal urgency to undergo CABG in the same hospital. Each patient contributed 1 count to the list size for each week that he or she remained on the wait list, except for the week of registration. Because CABG surgeries are confirmed 1 week in advance, patients who are to undergo surgery are considered removed from the wait list during the week before their admission date. We defined the number of direct admissions as the weekly count of CABG surgeries performed without wait-list registration.

### Statistical analysis

#### Primary outcome

In this study, the primary outcome was the death of patients awaiting CABG on a wait list referred to as wait-list deaths. The time on a wait list was computed as the number of calendar weeks from registration to surgery, death, or wait-list removal. The date of surgeon's request for booking the operating room serves as the date of registration on a wait list. The probability of remaining on the list after a certain time was estimated by the product-limit method [11]; wait-list times were treated as prospective observations that were monitored from registration to the patient's last week on the list. The log-rank test was used to compare the time to removal across the study groups [12]. The average weekly rate of wait-list deaths was determined by dividing the number of deaths by the sum of observed wait-list times.

#### Study variables

The wait-list size was categorized by clearance time; i.e., a hypothetical time within which the list could be cleared at the maximum weekly service capacity if there were no new arrivals [13]. We categorized wait-list size as either "1 month or less" or "more than 1 month" of clearance time. We chose 1 month as a cut-off, reasoning that registration on a wait list with a clearance time of 1 month or less permits undergoing surgery within the planned access time of 6 weeks for semi-urgent patients. In 3 of the 4 participating hospitals, which had a service capacity of performing 15 operations per week, a wait list of 59 or fewer patients corresponded to a clearance time of 1 month or less, and a wait list of 60 or more patients corresponded to a clearance time of more than 1 month. In the fourth hospital, which had a service capacity of performing 25 operations per week, a wait list of 99 or fewer patients corresponded to a clearance time of 1 month or less, and a list of 100 or more patients corresponded to a clearance time of more than 1 month. The weekly number of direct admissions was treated as a continuous variable.

#### Cumulative incidence for wait-list death

We used the cumulative incidence function (CIF) to characterize the time-dependent, marginal probability that pre-operative death occurs on or before a certain wait-list week. We interpreted the cumulative incidence for wait-list death as the proportion of patients who were to undergo CABG but died before surgery; a number that increased over wait-list time. The CIF for wait-list death is defined as the integration over time of the product of the weekly death rate and the probability of remaining on the list [14]. The CIF of wait-list death and its standard errors were estimated using non-parametric methods [15]. We used a 2-sample test to compare the CIFs between categories of wait-list clearance time [16].

#### Regression models

The effect of wait-list clearance time on the weekly death rate was estimated by means of discrete-time survival regressions that yield the odds ratio (OR) as a measure of the effect size [17]. We used discrete-time survival analysis because wait-list time is inherently discrete and is best measured by the number of weekly operating room schedules [13]. To test for differences in the CIF between list-size categories, we used competing-risk regression models based on pseudo-values of the CIF [18]. The clearance-time category was added as an indicator variable, with 1 denoting a clearance time of more than 1 month. The exponential of the regression coefficient for that variable gives the odds ratio of pre-operative deaths for category "more than 1 month" relative to category "1 month or less". Pseudo-values for the CIF for wait-list death were computed in the presence of surgery and other competing events at all distinct, observed event times. For each patient, the CIF pseudo-values corresponded to a series of binary variables equal to zero before and 1 at or after death in the absence of censoring. The CIF models were adjusted for subject-level correlation between pseudo-values using the generalized estimation equations. The working weight matrix was fixed and estimated as a product-moment correlation matrix among the pseudo-values. For the direct admissions, we interpret odds ratios as a change in the weekly odds of wait-list death associated with 1 additional surgery performed immediately after angiography.

#### Confounders

Multivariate analyses controlled for differences in patients' characteristics and significant confounders summarized in Table 1. Existing literature suggests that elderly patients are more likely to undergo revascularization as an urgent procedure [19]; smaller coronary vessel diameters may account for higher risk of adverse events in women [20]; co-existing medical conditions may delay open heart surgery [21]; and changes in practice or supplementary funds may reduce time to surgery [10]. We entered two indicator variables for three comorbidity categories, referent, no co-existing conditions, and 2 comparison categories: presenting with congestive heart failure, diabetes, chronic obstructive pulmonary disease, cancer or rheumatoid arthritis as suggested by Naylor and colleagues [22]; or presenting with other co-existing chronic conditions as defined in Romano and colleagues [23].

## Results

### Patients

Table 1 shows the distribution of wait-listed patients and direct admissions according to age, sex, calendar period, urgency for surgery, comorbid conditions, and wait-list clearance time at registration. In the group of patients who underwent surgery without registration on wait lists, the age distribution was similar to the listed patients, with the majority (68%) undergoing surgery between 60 and 79 years. Compared with the listed patients, the proportion of women (22%) was slightly higher. Differences between these two groups by urgency and coexisting medical conditions indicate that sicker patients were more likely to undergo operation without delay. For example, less than 6% of wait-listed patients were in urgent category compared with 51% for directly admitted. Similarly, almost 53% of wait-listed patients had no co-existing conditions, compared with only 11% in the other group. Wait lists with 1 month or less of clearance time were observed in all urgent patients and were more prevalent in semi-urgent than non-urgent patients (79.1% vs 44.7%, respectively).

**Table 1:** Characteristics of 8,966 wait-listed patients and 10,467 directly admitted patients isolated coronary artery bypass surgery in British Columbia 1991–2001

**Characteristic**	**Wait-Listed patients N(%)**		**Direct admissions N(%)**	
Age group (yr)				
<50 yr	717	(8.0)	808	(7.7)
50–59 yr	1966	(21.9)	2082	(19.9)
60–69 yr	3425	(38.2)	3689	(35.2)
70–79 yr	2676	(29.8)	3509	(33.5)
≥80 yr	182	(2.0)	379	(3.6)
Sex				
Women	1581	(17.6)	2313	(22.1)
Men	7385	(82.4)	8154	(77.9)
Period of registration/surgery				
1991–1992	1675	(18.7)	1770	(16.9)
1993–1994	1859	(20.7)	1526	(14.6)
1995–1996	1867	(20.8)	1686	(16.1)
1997–1998	1853	(20.7)	1997	(19.1)
1999–2000	1712	(19.1)	2454	(23.4)
2001			1034	(9.9)
Urgency at registration/surgery				
Urgent	515	(5.7)	5353	(51.1)
Semi-urgent	6444	(71.9)	4536	(43.3)
Non-urgent	2007	(22.4)	523	(5.0)
Not provided			55	(0.5)
Comorbidity at registration/surgery				
Major conditions^†^	1930	(21.5)	4040	(38.6)
Other conditions^‡^	2304	(25.7)	5268	(50.3)
None	4732	(52.8)	1159	(11.1)
Wait-list clearance time				
1 month or less	6512	(72.6)		
more than 1 month	2454	(27.4)		

^†^congestive heart failure, diabetes, chronic obstructive pulmonary disease, rheumatoid arthritis, cancer

^‡^peripheral vascular disease, cerebrovascular disease, dementia, peptic ulcer disease, hemiplegia, renal disease, or liver disease

### Outcomes of registration for CABG

By 52 weeks on the list, 7724 (86.1%) patients had undergone surgery, and 767 (8.6%) had been removed without surgery for various reasons such as having died while awaiting surgery (92 patients), continuing medical treatment (176), refusal of surgery (188), having been accepted for surgery by another surgeon or hospital (99), having undergone another type of surgery (23), or other reasons (189). Death certificates were available for 87 of the 92 patients who died while awaiting operation, and 5 sudden deaths were reported by the participating hospitals. Of the 515 urgent patients, 98 (19.0%) were downgraded to the semi-urgent or non-urgent category at the time of surgery.

More than 10% (258) of non-urgent patients and about 5% (212) of semi-urgent patients were still on the wait lists at 52 weeks. Five patients in the urgent group had calculated wait times of more than 52 weeks. One of those patients was eventually removed by request, the urgency for surgery was downgraded in 2 patients, and the reason for the delay in surgery was unknown in 2 patients. In total, 254 (2.8%) patients were removed from the wait lists for CABG after being deemed unfit for surgery.

**Figure 1:**
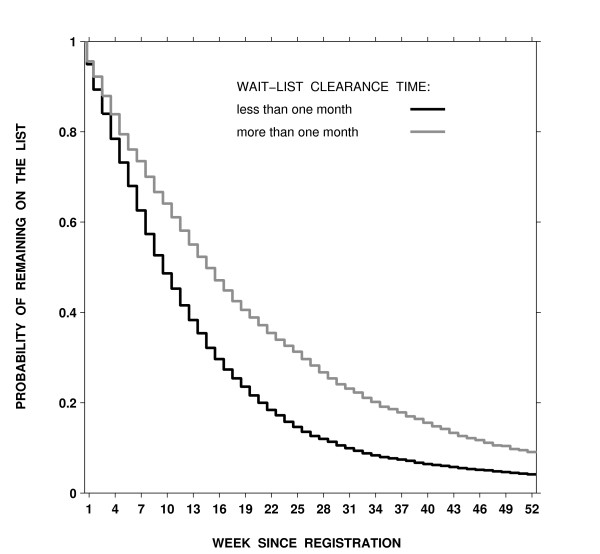
Estimated probability of remaining on a coronary-artery bypass grafting wait list by the number of weeks since registration and wait-list clearance times in semi-urgent and non-urgent groups combined.

Figure 1 shows the estimated probability of remaining on the list by week since registration and wait-list clearance time. Lists with longer clearance times were associated with longer wait times (the log-rank test = 266.4, df = 1, p < 0.001). When a clearance time was 1 month or less, 75%, 50%, and 25% of patients remained on wait lists after 4, 9, and 18 weeks, respectively. For a clearance time of more than 1 month, 75%, 50%, and 25% of patients remained on the list after 6, 14, and 29 weeks, respectively.

### Death rates by clearance time

The effect of wait-list clearance time was studied in semi-urgent and non-urgent patients because all urgent patients fell in one clearance time category. There were 49 wait-list deaths over 84,710 patient-weeks of follow-up in category "1 month or less", and 39 deaths over 49,219 patient-weeks in category "more than 1 month". The observed average death rate was higher in category "more than 1 month" than in category "1 month or less", 0.79 (95% confidence interval [CI] 0.54–1.04) vs. 0.58 (95% CI 0.42–0.74) per 1000 patient-weeks. After adjustment for age, sex, urgency for surgery, calendar period, co-existing conditions, and weeks on the list, the weekly odds of wait-list death were 1.6 higher greater for a longer clearance time, the adjusted OR = 1.60 (95% CI 1.01–2.53). In semi-urgent and non-urgent groups, the product of the average death rates and weeks on the wait list served as a good approximation for the cumulative hazards, suggesting that the hazard functions for wait-list death were constant over wait-list time.

### Cumulative incidence for wait-list death

**Figure 2:**
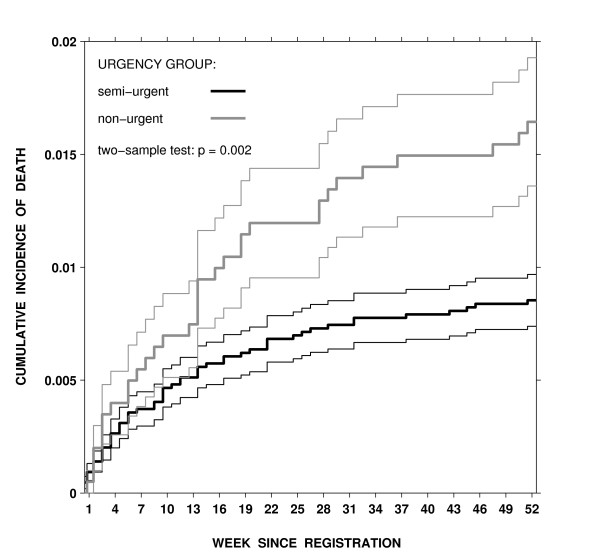
Estimated cumulative incidence for death on the wait list by the number of weeks since registration and urgency group, thin lines represent standard errors for the cumulative incidence estimate for each week

Figure 2 shows the estimated cumulative incidence for wait-list death by clearance-time categories in semi-urgent and non-urgent patients combined. The observed (unadjusted) proportion of wait-list deaths by 52 weeks was lower in category "1 month or less" than in category "more than 1 month" (0.8% [49 deaths] vs. 1.6% [39 deaths], Gray's 2-sample test = 10.1, df = 1, *P *< 0.005). Higher weekly death rates and longer waits in the group with a clearance time of more than 1 month contributed to the differences in the cumulative incidence of wait-list death between the groups studied. After adjustment for age, sex, urgency for surgery, calendar period, co-existing conditions, and weeks on the list, the effect of wait-list size at registration remained significant. The odds of wait-list death were 64% higher in patients on a list with a clearance time of more than 1 month than in those on a list with a clearance time of 1 month or less, the adjusted OR = 1.64 (95%CI, 1.02–2.63), Table 2. As expected, the urgency for surgery had a major influence on the cumulative incidence of wait-list death as well. Non-urgent patients had a higher cumulative incidence of pre-operative death than did semi-urgent patients for almost all weeks on the list (Gray's 2-sample test = 9.3, df = 1, *P <*0.001). After controlling for confounders, the difference between urgency groups remained significant and independent from the list-size effect, the adjusted OR = 1.69 (95%CI, 1.05–2.74). Direct admissions did not alter the odds of death for semi-urgent and non-urgent patients.

**Table 2:** Association between urgency, wait-list clearance times and cumulative incidence for death on the wait list as measured by odds ratios derived from discrete-time survival regression models

**Effect**	**Unadjusted OR(95% CI)**	Adjusted OR*(95% CI)
non-urgent vs semi-urgent	1.61(1.00, 2.59)	1.69(1.05, 2.74)
clearance time of 1 month or less	1.00	1.00
clearance time of more than 1 month	1.67(1.05, 2.66)	1.64(1.02, 2.63)
direct admission^†^	--	1.00(1.00, 1.00)

*Adjusted for age, sex, comorbidity, calendar period, and week on the list

†Associated with one additional surgery performed without wait-list registration

## Discussion

We examined the relationship between the length of the wait list at the time of registration for CABG and the risk of death before surgery in patients awaiting that operation on any of multiple wait lists in a health system in which all medically necessary services are publicly funded. Using records from the provincial population-based registry of patients identified as needing surgical revascularization, we compared the cumulative incidence for wait-list death between the two categories of wait-list size according to a clearance time. The list size was a simple count of patients with higher or equal surgical priority who were on a wait list at the time of registration of a new patient. Out of 88 wait-list deaths that occurred in the two less urgent groups, 44 deaths in semi-urgent and 15 deaths in non-urgent groups were related to cardiovascular disease. We report on all-cause mortality because the accuracy of death certificate codes is a concern in this analysis; using all-cause mortality could not have induced bias in the results [24].

Our results show that wait-list size is associated with the probability that a semi-urgent or non-urgent patient would die before surgery by a certain wait-list week. The patients registered on a list with a clearance time of more than 1 month had 60% higher weekly death rate after adjustment than those on a list with a clearance time of 1 month or less. Longer wait times (p < 0.001) and a higher death rate contributed to a higher cumulative incidence for wait-list death in the patients registered on a list with a clearance time of more than 1 month, the adjusted OR = 1.64 (95% CI 1.02–2.63). The number of patients who underwent CABG without having been registered on a wait list in the same hospital exerted no independent effect.

Other investigators concerned with delay in treatment for patients who require a CABG have reported on the impact of patient prioritization [25,26], risks of delayed treatment [1,2,27], and the worsening symptoms and morbidity associated with a long wait for surgery [3,28]. In quantifying the risk of adverse events on wait lists for CABG surgery, the Kaplan-Meier method is often used to estimate the cumulative probability of the occurrence of an event by certain time after registration for surgery [3,28,29]. It has been found, however, that the complement of Kaplan-Meier estimator overestimates the proportion of the event in the competing risks setting [30]. Because patients on a wait list are subject to competing events such as surgery, death, or removal from the wait list for other reasons, the Kaplan-Meier method produces probability estimates that are only valid in a hypothetical situation in which all competing risks are removed before the patient's death without altering the risk of death [31]. Without the assumption of independent competing events, the Kaplan-Meier method is not valid and should not be used [32]. However, the independence of wait outcomes cannot be verified from data and may not be realistic, because the low proportion of wait-list deaths may indicate either a low risk of death or a high rate of surgery. Appropriate statistical instruments include the CIF that can be estimated without the independence assumption for competing events. The CIF describes the time-dependent marginal probability that pre-operative death occurs on or before a certain time of registration on a wait list after the probability of surviving multiple competing events has been considered [14,33,34]. Pepe and Mori argued that the CIF is a more accurate and comprehensive summary of the risk of death in a competing-risks setting than are death rates or cumulative hazards, which cannot be translated to the probability of death [15].

Misclassification of the recorded urgency for treatment is a concern in this analysis. Retrieved from the registry, the urgency category is a composite variable that is based on a variety of clinical factors. No audit was performed to evaluate the quality of those records. The observation that higher priority patients were more likely to undergo CABG via direct admission indicates that the degree of misclassification of priority was likely small. Another concern is that in some patients, the urgency for surgery was reclassified at the time of surgery. However, the timing of changes in urgency was not recorded.

## Conclusion

The contribution of this paper is two-fold. First, the cumulative incidence for wait-list death in relation to wait-list size at the time of registration for CABG, to our knowledge, has not been reported previously. We found that long wait lists are associated with increased probability that a patient dies before surgery after accounting for the surgery rate in semi-urgent and non-urgent patients. Second, physicians who advise patients to undergo revascularization with a cardiac surgeon can use our results to consider the risk of pre-surgical death that is associated with the current length of wait list of the surgeon.

## Authors' contributions

BS conceived the study concept and design, participated in analysis and interpretation, and drafted the manuscript. LK performed statistical analysis and drafted the manuscript. AL participated in data acquisition and critically revised the manuscript. RH participated in data acquisition and critically revised the manuscript. All authors read and approved the final manuscript.

**Figure 3:**
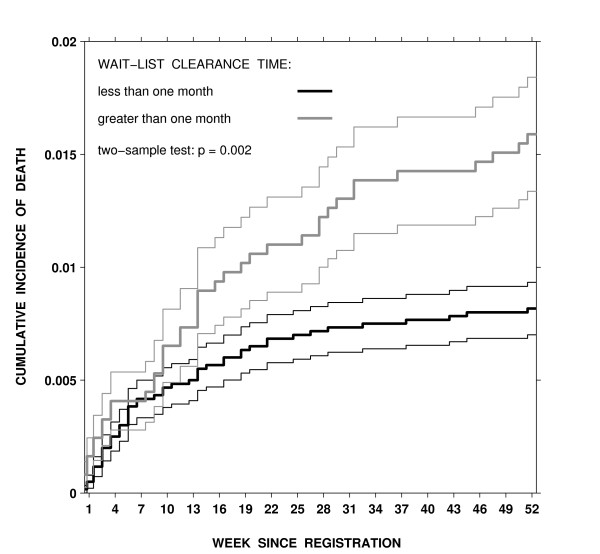
Estimated cumulative incidence for death on the wait list by the number of weeks since registration and wait-list clearance times in semi-urgent and non-urgent groups combined, thin lines represent standard errors for the cumulative incidence estimate for each week
